# The effect of the combined use of complex decongestive therapy with electrotherapy modalities for the treatment of breast cancer-related lymphedema: a randomized clinical trial

**DOI:** 10.1186/s12891-022-05780-1

**Published:** 2022-09-03

**Authors:** Mahboobeh Hemmati, Zahra Rojhani-Shirazi, Zeinab Sadat Zakeri, Majid Akrami, Nasrin Salehi Dehno

**Affiliations:** 1 Student Research Committee, Shiraz University of Medical Sciences, Shiraz, Iran; 2 Physical Therapy Department, School of Rehabilitation Sciences, Shiraz University of Medical Sciences, Abiverdi 1, Chamran Blvd, P.O. Box: 71345-1733, Shiraz, Iran; 3 Rehabilitation Sciences Research Center, Shiraz University of Medical Sciences, Shiraz, Iran; 4 Shiraz Lymphedema Clinic, Breast Diseases Research Center, Shiraz University of Medical Science, Shiraz, Iran

**Keywords:** Breast cancer, Complex decongestive therapy, Disability, Electrotherapy, Lymphedema, Pain

## Abstract

**Background:**

We evaluated the effects of combined complex decongestive therapy (CDT) with electrotherapy modalities (ultrasound and faradic currents) in patients with breast cancer-related lymphedema (BCRL), investigating upper extremity circumference, volume, pain, and functional disability.

**Methods:**

Thirty-nine patients with unilateral BCRL were randomly allocated to three groups (*n* = 13) as the following: The control group received CDT, the ultrasound group received CDT and therapeutic ultrasound, and the faradic group received CDT and faradic current. All the participants underwent treatment for 10 sessions. The outcomes including volume, circumference (measured at five points), pain intensity, and functional disability of the affected upper extremity were evaluated at baseline and after the treatment.

**Results:**

Following the treatment, an improvement was noted in lymphedema volume, pain, and functional disability in all the three groups and there was a significant difference between the groups (*P* < 0.05). However, changes in limb circumference at the end of the treatment were not significantly different among the three groups in any sites (*P* > 0.05).

**Conclusion:**

The combination of electrotherapy modalities, faradic current or ultrasound, with CDT can result in a greater reduction in lymphedema volume, pain, and functional disability in patients with BCRL.

**Trial registration:**

IRCT, IRCT201310292391N14, registered 03/01/2016.

## Introduction

Breast cancer is the most prevalent malignancy and the leading cause of cancer-related deaths among women worldwide [1]. Various types of treatments, including surgery, chemotherapy, and radiotherapy, have been developed for its treatment. Upper extremities lymphedema is a common physical complication after breast cancer treatment, which is characterized by persistent tissue swelling in the extremity due to abnormal accumulation of lymph in tissues [2]. The pooled incidence of breast cancer-related lymphedema (BCRL) is estimated to be about 17% [3]. BCRL is known to be a chronic and progressive condition causing considerable functional and psychological disturbance [4, 5]. Although it cannot be fully treated, the current treatments can manage the condition and slow down or prevent its progression [6].

Physical therapy is essential for the treatment of lymphedema and a variety of interventions, including complex decongestive therapy (CDT), ultrasound, cryotherapy, laser therapy, electrotherapy, resistive exercise, and kinesio taping have been proposed to minimize its associated complications by reducing upper extremity swelling [7, 8]. CDT is the most common treatment, which involves manual lymphatic drainage (MLD), compression therapy, remedial exercise, and skin care [9]. Even though CDT has been proven as the most widely used treatment for lymphedema [10–12], it has been reported that combined techniques contribute to a more complete and efficient treatment [13]. Effective treatment of lymphedema in patients with breast cancer is of great importance and can enhance their quality of life [14, 15]. Several studies have reported more improvement following combined techniques [16–19]. Koo et al. reported a more significant improvement after combination of heperbaric oxygen therapy with CDT in patients with BCRL [17]. Lee et al. concluded that adding shockwave therapy to the CDT resulted in further improvement in upper limb circumference and volume and skin thickness in patients with BCRL [18]. Pekyavas et al. also found that combining CDT and kinesio taping was more effective on volume reduction and quality of life [19].

To date, no studies have investigated the effectiveness of interventions featuring combinations of electrotherapy modalities, such as ultrasound therapy and faradic current with CTD in patients with BCRL. The present study was therefore designed to evaluate the effect of combining CDT and ultrasound or faradic current on swelling, pain, and functional disability of upper extremity in patients with BCRL. The hypothesis is that in these patients, combination of CDT with ultrasound or faradic current would improve the outcomes more significantly compared with CDT alone.

## Materials and methods

### Study design and participants

This study was a single-blinded randomized controlled trial with 3 parallel groups. 39 female patients diagnosed with BCRL, who were referred to Shahid Mottahari Therapeutic Center in affiliated with Shiraz University of Medical Sciences were recruited in the present work. The patients were informed about the study and written informed consent was obtained from all of them. The study protocol followed the ethical principles of the Declaration of Helsinki, and was approved by the Ethics Committee of the Shiraz University of Medical Sciences (protocol number: ir.sums.rec.1394.167) and in the Iranian Registry of Clinical Trials (IRCT201310292391N14, 03/01/2016).

The inclusion criteria were being above 18 years of age, a diagnosis of unilateral breast cancer, history of surgery, chemotherapy or radiotherapy, > 2 cm difference in circumference, and /or > 10% difference in volume between the affected and unaffected upper extremities [20]. The exclusion criteria were the primary lymphedema or bilateral lymphedema, active cancer, skin infection or radiotherapy burn in the affected extremity, rheumatic diseases, renal failure, congestive heart failure, ulcers in the affected arm, arterial or venous disease, and uncontrolled hypertension. The patients who received CDT or other interventions for lymphedema within 12 months were also excluded.

### Randomization and blinding

The participants were randomly assigned to three treatment groups using the block permutation method as the following: control group (receiving CDT therapy), ultrasound group (receiving CDT therapy and therapeutic ultrasound), and faradic group (receiving CDT therapy and faradic current). The allocation concealment was carried out using sequentially numbered, sealed, and opaque envelops. The procedure was conducted by a researcher who did not play any role in the treatment or assessment of the participants. All the evaluations were performed by the same physician who was blinded to group assignment and all the intervention protocols. Blinding of the treating physiotherapist and patients was impossible on account of the nature of the intervention. Due to the lack of similar studies, we conducted a pilot trial with a sample size of 39 patients, with each group comprising 13 subjects.

### Intervention

The patients in all the three groups received 10 treatment sessions (five sessions per week) by an experienced physiotherapist.

They all underwent a standard protocol of CDT comprising MLD, compression therapy with a short stretch bandage, skin care, and lymphedema exercises. CDT was performed for 1 h a day. MLD was performed for 30 min in a proximal to distal direction from the affected extremity toward the unaffected side with light skin massage [21]. Multi-layer compression bandages were used for compression, which were changed daily except the weekends. The patients were asked to keep the bandaging on the arm for 23 h a day. All the subjects were educated on proper skin care, such as skin hygiene, applying moisturizer daily, and avoiding excessive heat and trauma. They were also given a standard lymphedema exercise program, including breathing exercises, neck and shoulder range of motion, and stretching exercises, in order to help facilitate lymphatic flow [21].

The patients in the ultrasound group were treated with CDT and 1 MHz, 2 W/cm2 pulsed ultrasound via a therapeutic ultrasound generator (Novin, 215X, joint product of Iran and England). Ultrasound was applied on the midpoint of the line between the elbow joint and the acromion, biceps lateral tendon in the elbow joint, midpoint of the line between the olecranon and ulnar styloid (on the anterior and posterior surfaces of the forearm), and the anterior part of the wrist, for 3 min in each area.

The patients in the faradic group received CDT and faradic current utilizing a stimulator (model 710L, Novin, Iran). The faradism under pressure was given at a frequency of 30 Hz, duration of 300 µs, interval of 2 s and off time of 5 s on the flexor and extensor forearm muscles of the affected upper extremity (10 min on each surface). The electrodes were held in place with elastic bandages wrapped in a distal to proximal direction on the upper limb*.*

The length of the treatment session for the control group was 1 h (CDT therapy only) while this length for the ultrasound group (CDT therapy and therapeutic ultrasound) was about 1 h and 15 min and for the faradic group (CDT therapy and faradic current) was about 1 h and 20 min.

### Outcome measures

All the measurements were undertaken prior to the treatment and at the end of 10 treatment sessions. The primary outcomes were extremity circumference and volume and the secondary ones included pain and shoulder disability.

Using a tape measure, circumference was measured in wrist, the middle of the forearm (midpoint between the wrist and elbow), elbow, the middle of the upper arm (midpoint between the olecranon and acromion), and 65% of the distance from the olecranon to the acromion) with the patient seated with their arms relaxed by their side and elbows straight [22].

The volume of the arm was measured via water displacement method as the gold standard method for volumetric measurements and determining volume reduction in patients with lymphedema [23]. The utilized volumeter consisted of a pair of specially constructed cylindrical plexi-glass tanks, each with two drainage taps [24]. Internal tank measured 70.0 cm in height by 21.0 cm in diameter. The external tank measured 60 cm in height and 31.0 cm in diameter. The section area of the internal tank was 330.0 cm^2^. The internal tank was filled with water to a height of 70.0 cm. The outer wall of the external tank was marked in centimeters and millimeters to measure the height of the water that overflowed from the inner tank. The patient stood next to the device and immersed her straightened healthy hand and arm into the inner tank up to a point 15 cm above the olecranon [24]. The height of the displaced water that spilled into the outer tank was recorded in centimeters. The patient then removed her healthy upper limb and immerses her affected hand and arm into the internal tank, and the height of the displaced water was again recorded. The difference between the two measurements was multiplied by the section area of the inner tank to calculate the volume of water in milliliters displaced by the arm with lymphedema compared to the unaffected limb [24].

Pain intensity was assessed using the numerical rating pain scale (NRPS) ranging from 0 = no pain to 10 = worst imaginable pain [25]. The patients were asked to report the level of pain intensity experienced during the preceding week.

The functional disability of the affected upper extremity was assessed through the disabilities of the arm, shoulder, and hand (DASH) questionnaire, which evaluates physical function and symptoms associated with limitations of arm, shoulder, and hand. The total DASH score ranges from 0 to 100; higher scores indicate more functional disability [26]. In this study, the Persian version of the DASH questionnaire was used, which was previously shown to be reliable and valid for the Iranian population with upper extremity disorders [27].

### Statistical analyses

The continuous data were tested for normal distribution using the Kolmogorov–Smirnov. Nonparametric Wilcoxon signed-rank test was employed to compare the variables before and after the treatment in each group, and the Kruskal–Wallis test was used to compare the results between the groups. One there was a significant between-group difference, Post hoc analysis with Bonferroni correction was used for pairwise comparison. The analyses were carried out with SPSS version 23 (IBM statistics, New York, USA) and the values of *P* < 0.05 were considered to be statistically significant.

## Results

**Fig. 1:**
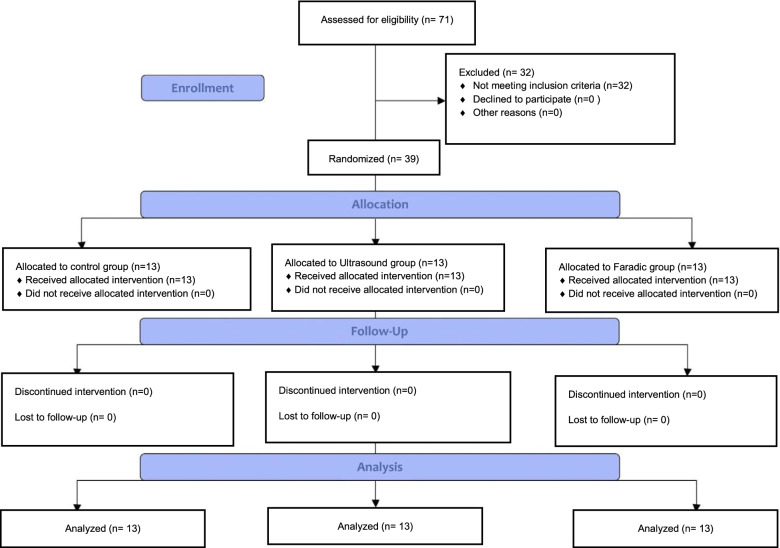
Flow chart of study implementation. Control group: received complex decongestive therapy; Ultrasound group: received complex decongestive therapy and therapeutic ultrasound; Faradic group: received complex decongestive therapy and faradic current

Figure 1 represents the CONSORT flow chart. A total of 78 females were screened among the patients referred to lymphedema rehabilitation unit for the treatment of lymphedema, 39 of whom were enrolled in this study. The demographic and clinical characteristics of the patients in the three groups did not differ significantly (Table 1). Also, no significant differences between groups were observed at baseline in any of outcome measures (*p* > 0.05).

**Table 1:** Demographic and clinical characteristics of participants

variables	Control group (*n* = 13)	Faradic group (*n* = 13)	Ultrasound group (*n* = 13)	*P*
Age (years)	49.13 ± 10.50	48.96 ± 10.12	49.32 ± 10.15	0.82
Height(cm)	158.65 ± 5.75	159.73 ± 5.50	160.32 ± 5.60	0.51
Weight (kg)	65.56 ± 7.66	62.78 ± 9.29	64.76 ± 8.5	0.20
Body mass index (kg/m^2^)	23.71 ± 3.26	24.55 ± 3.11	23.33 ± 3.33	0.370
Type of surgery				
Breast conserving	2	5	3	0.390
Mastectomy	11	8	10	
Side of lymphedema				
Right	5	6	7	0.734
Left	8	7	6	
Duration of lymphedema (months)	21.53 ± 7.98	29.69 ± 10.38	25.46 ± 12.12	0.138

Values are mean ± SD or numbers

Circumference of the affected limb at wrist did not differ significantly between the baseline and the end of the treatment in the control group. However, it was significantly reduced at the end of the treatment in the Faradic (*P* = 0.009) and ultrasound therapy (*P* = 0.014) groups. The circumference in the middle of forearm was significantly reduced at the end of the treatment in all the three groups (Control: *P* = 0.005, Faradic: *P* = 0.019, Ultrasound: *P* = 0.004). The circumference in elbow significantly decreased at the end of the treatment in all the three groups (Control: *P* = 0.02, Faradic: *P* = 0.01, Ultrasound: *P* = 0.016). Additionally, there was a significant decrease in circumference in the middle of arm at the end of the treatment in all the three groups (Control: *P* = 0.019, Faradic: *P* = 0.003, Ultrasound: *P* = 0.028). However, circumference in 65% of the distance from the olecranon to the acromion was significantly reduced only in the Faradic group (Control: *P* = 0.45, Faradic: *P* = 0.023, Ultrasound: *P* = 0.373) (Table 2). The Kruskal–Wallis test showed that the changes in limb circumference at the end of the treatment were not significantly different among the three groups in any sites (wrist *P* = 0.062, middle of forearm: *P* = 0.12, elbow: *P* = 0.215, middle of arm: *P* = 0.333, 65% of the distance from the olecranon to the acromion: *P* = 0.31) (Table 2).

**Table 2:** Comparison of changes in circumference, volume, pain and disability within and between groups

Variable	Group	Before treatment	After treatment	Change	*P* within groups	*P* Between groups
Circumference at wrist (cm)	Control	17.70 (14.80 to 28.80)	17.80 (14.90 to 20.30)	0.1 (-10.80 to 1.4)	0.97	0.062
	Faradic	17.60 (15.30 to 25.30)	16.70 (14.90 to 19.20)	-0.6 (-6.9 to 0.5)	0.009	
	Ultrasound	17.80 (15.90 to 21.50)	17.30 (15.90 to 19.10)	-1 (-3.8 to 0.2)	0.014	
Circumference at middle of forearm (cm)	Control	26.40 (20.70 to 31.40)	25.50 (20.20 to 29.80)	-1.2 (-3.2 to 0.7)	0.005	0.12
	Faradic	25.30 (18.70 to 37)	24.25 (18.80 to 28.50)	-1.9 ( -8.5 to 2.7)	0.019	
	Ultrasound	27.20 (22.40 to35.30)	23.90 (21.80 to 28.30)	-2.5 (-9 to 0.5)	0.004	
Circumference at elbow (cm)	Control	27.00 (22.30 to 31.80)	26.10 (21.70 to 31.50)	-1.3 (-3.1 to 1.3)	0.021	0.215
	Faradic	26.20 (21.00 to 36.00)	25.30 (21.00 to 30.50)	-0.7 (-6.1 to 1.2)	0.01	
	Ultrasound	29.80 (24.80 to 38.10)	27.80 (22.90 to 30.50)	-2.4 (-7.6 to 0.5)	0.016	
Circumference at middle of arm (cm)	Control	30.20 (25.80 to 34.90)	28.70 (25.30 to 34.30)	-0.8 (-2.4 to 0.7)	0.019	0.333
	Faradic	31.80 (23.40 to 49.00)	30.20 (23.40 to 34.20)	-1 (-14.8 to 0.0)	0.003	
	Ultrasound	33.00 (27.80 to 43.90)	30.90 (24.00 to 36.20)	-2 (-10.50 to 0.5)	.0.028	
Circumference at 65% of distance from olecranon to acromion (cm)	Control	32.20 (26.50 to 35.90)	31.8 (26.70 to 35.30)	-0.1 (-1.3 to -1.4)	0.455	0.31
	Faradic	32.70 (24.80 to 36.30)	32.10 (24.20 to 35.30)	-0.6 (-1.7 to 0.9)	0.023	
	Ultrasound	32.30 (28.40 to 40.60)	31.80 (22.50 to 35.8)	0.0 ( -10.5 to 1.5)	0.373	
Volume difference (ml)	Control	690 (0 to 1170)	570 (0 to 990)	-120 (-210 to 0.00)	0.002	0.005
	Faradic	450 (150 to 3570)	270 (60 to 1320)	-210 (-2250 to -60)	0.001	
	Ultrasound	780 (60 to 2370)	540 (60 to 840)	-270 (-1770 to 30)	0.004	
Pain intensity (score)	Control	10 (8 to10)	7 (6 to 8)	-3 (-4 to -2)	0.001	0.001
	Faradic	10 (8 to 10)	2 (2 to 4)	-8 ( -8 to -6)	0.001	
	Ultrasound	10 (8 to 10)	2 (1 to 4)	-8 (-9 to -6)	0.003	
Functional disability (score)	Control	83.33 (66.67 to 91.67)	58.33 (50 to 79.17)	-18.33 (-25 to -12.50)	0.001	0.001
	Faradic	83.33 (58.33 to 91.67)	25.83 (16.67 to 35)	-56.66 (-62.5 to -33.33)	0.001	
	Ultrasound	83.33 (75 to 100)	25 (19.17 to 43.33)	-56.6 (-60.83 to -51.67)	0.003	

Values are median except those in parenthesis, which are minimum to maximum

The volume differences between the two upper limbs were significantly reduced following the treatment in all the groups (Control: *P* = 0.002, Faradic: *P* = 0.001, Ultrasound: *P* = 0.004). The Kruskal–Wallis test revealed that the changes in the volume at the end of the treatment were significantly different among the three groups (*P* = 0.005) (Table 2). Pairwise comparison showed that the difference between the faradic and control groups was not significant (*P* = 0.084) whereas that between the ultrasound group and control group was statistically significant (*P* = 0.005). No significant differences were found between the faradic and ultrasound groups (*P* = 0.916) (Table 3).

**Table 3:** Pairwise comparison of changes in volume, pain and functional disability between groups

Variable	Group	*P* value
Volume difference (ml)	Control – Faradic	0.084
	Control – Ultrasound	0.005
	Ultrasound – Faradic	0.916
Pain severity (score)	Control – Faradic	0.001
	Control – Ultrasound	0.001
	Ultrasound – Faradic	0.89
Functional activity (score)	Control – Faradic	0.001
	Control – Ultrasound	0.001
	Ultrasound – Faradic	0.622

Pain severity score declined significantly at the end of the treatment. This reduction was significant in the all three groups (Control: *P* = 0.001, Faradic: *P* = 0.001, Ultrasound: *P* = 0.003). The Kruskal–Wallis test implied that the changes in pain severity at the end of the treatment were significantly different among all the three groups (*P* = 0.001) (Table 2). Pairwise comparison showed a significant difference in pain severity between the faradic and the control groups (*P* = 0.001) and between the ultrasound and control groups (*P* = 0.001). Meanwhile, no significant differences were seen between the faradic and ultrasound groups (*P* = 0.89) (Table 3).

Functional disability in upper extremity was improved at the end of the treatment in all the groups (Control: *P* = 0.001, Faradic: *P* = 0.001, Ultrasound: *P* = 0.003) (Table 2). This improvement was greater in the faradic and ultrasound groups compared with that in the control group. The Kruskal–Wallis test demonstrated a significant difference concerning the changes in functional disability at the end of the treatment among all the three groups (*P* = 0.001) (Table 2). There was a significant difference between the faradic and the control groups (*P* = 0.001), and between the ultrasound and control groups (*P* = 0.001). However, the difference between the faradic and ultrasound groups was not significant (*P* = 0.622) (Table 3).

## Discussion

The current study investigated the effectiveness of combined CDT with electrotherapy modalities in BCRL treatment. Our findings demonstrated greater reduction in lymphedema volume, pain, and functional disability in the two groups submitted to a combination of CDT with ultrasound or CDT with faradic current. However, we did not observe any significant differences in arm circumference at any measured points among the groups.

Lymphedema following breast cancer treatment is a chronic lifelong complication caused by reduced transport capacity of the lymph system, which significantly affects functionality of the upper extremity and quality of life [28]. Our results are in agreement with those of previous papers, demonstrating that CDT is effective in treating lymphedema associated with breast cancer [29–31]. CDT therapy is currently used as the first-line therapy for lymphedema. The effect of this therapy is largely influenced by factors of professional specialization, patient education, and compliance. Depending on the condition, extensive intervention period may be required, which may lead to a lower patient compliance while increasing the cost burden [32, 33]. Nonetheless, the effectiveness of CDT on the symptoms associated with lymphedema remains controversial [9, 34].

Due to these limitations, additional treatment strategies need to be considered in order to optimize the treatment efficiency. In the present research, we found further effectiveness by combining CDT with ultrasound or faradic in the treatment of symptoms related to the BCRL. The parameters used herein for faradic current can trigger muscle contraction, which could contribute to favorable clinical results. Electrical stimulation reduces edema by increasing muscle contraction, which results in increased lymph flow and blood flow. Muscle contraction favors the removal of intercellular proteins; therefore, stimulating muscle contraction may be the most effective way to increase blood flow in muscles. There is evidence that blood flow can increase up to 30 folds during rhythmic muscle contractions. In addition, muscle exercises improve revascularization in muscles [35].

With respect to the effect of ultrasound on lymphedema, therapeutic ultrasound generates micro-massage flow through wave propagation at a cellular level with slight heat (a Joule effect), which modifies the microcirculation and cell metabolism. It also produces small local stress on the cell membrane and increases cell membrane permeability, which in turn, can improve lymph flow and reduce hardness of the fibrous tissue that appears after surgery [35, 36]. The results of a study, in which ultrasound was applied to the patients with lymphedema who had undergone breast cancer surgery, demonstrated a significant reduction in arm swelling, pain, and hardness in the arms [36].

Several studies, in line with the present findings, have indicated further improvement following combined therapy in patients with BCRL. Bok et al. investigated the effectiveness of CDT and progressive resistive exercise on patients with BCRL and observed a significant reduction in the subcutaneous tissue and circumference of affected upper extremity in the CDT and progressive resisted group [37]. In another study, Corum et al. found that the CDT combined with resistance exercise was effective in reducing circumference, volume, pain, and functional disability and improved grip strength and quality of life [38]. Lee et al. also reported that shockwave therapy with CDT can be more effective in improving circumference, volume, and skin thickness [18].

There are some limitations to this study which need to be considered. Primarily, our convenience sample included the patients from a single center, thereby limiting the ability to generalize the results to all the patients with the same condition. Secondly, since the patients were referred for treatment, ethical reasons did not allow us to have a control group without any types of therapy or a placebo group. Thirdly, a relatively small number of subjects may have partially affected the outcome. Finally, this study was designed to determine the treatment effects after 10 sessions of treatment with no further follow-ups. Further studies with a larger sample size and long-term follow-up are required to confirm the present results.

## Conclusion

The current paper shed light on the fact that in patients with BCRL, a combination of CDT with electrotherapy modalities results in a greater improvement in lymphedema volume, pain, and functional disability in comparison with CDT alone.

## Data Availability

The data file of this study is available to the corresponding author and can be made available to anyone upon reasonable request.

## Abbreviations

BCRL: Breast cancer-related lymphedema
CDT: Complex decongestive therapy
MLD: Manual lymphatic drainage
DASH: Disabilities of the arm, shoulder, and hand
